# Potential causes of black-stained peritoneal dialysis tubing: an analysis from nurse practitioner’s prospect

**DOI:** 10.1186/1756-0500-7-434

**Published:** 2014-07-06

**Authors:** Krit Pongpirul, Wannarat Amornnimit Pongpirul, Talerngsak Kanjanabuch

**Affiliations:** 1 Department of Preventive and Social Medicine, Faculty of Medicine, Chulalongkorn University, 1873 Rama IV Rd, 10330 Pathumwan, Bangkok, Thailand; 2 Department of International Health, Johns Hopkins Bloomberg School of Public Health, 21205 Baltimore, MD, USA; 3 Bumrungrad International Hospital, Bangkok, Thailand; 4 Department of Internal Medicine, Faculty of Medicine, Chulalongkorn University, 1873 Rama IV Rd, 10330 Pathumwan, Bangkok, Thailand

**Keywords:** Contamination, Delphi, Peritoneal dialysis, Nurse practitioner

## Abstract

**Background:**

Continuous Ambulatory Peritoneal dialysis (CAPD) has been promoted to be the main method of treatment for Thai End-Stage Renal Disease (ESRD) patients; however, a national survey of dialysis centers reported an annual incidence of black-stained particle of 57.6 per 1,000 CAPD cases. The objective of this study was to identify potential causes of the stain in the nurse practitioners’ prospect.

**Findings:**

This study applied three-round Delphi technique. In the first round, the questionnaire was sent to 127 nurses in all dialysis centers. Their responses were analyzed to come up with an anonymous summary, which was presented in the second and third round of the survey among 80 and 200 nurses. The response rates of the three rounds of Delphi were 57.5%, 81.3%, and 75.0%, respectively. Nurses consistently believed that the contamination was caused by spilled-out povidone-iodine solution during transfer set change. Other potential causes were previous peritonitis, inadequate dialysis, low serum albumin, transfer set soaking with antiseptics, patient history of diabetes, dressing technique, and existence of dry abdomen period.

**Conclusions:**

Black-stained particle is a common contamination of dialysis tube in CAPD patients. This study proposed some potential determinants, most of which were relevant to care process.

## Background

Continuous Ambulatory Peritoneal dialysis (CAPD) is the main method of treatment for Thai End-Stage Renal Disease (ESRD) patients under the “PD First” Policy of the Universal Coverage (UC) scheme [1]. More than 12,000 ESRD patients have registered for PD modality with participating PD centers under the UC scheme [1,2].

In 2008, the Tenckhoff tube and transfer set of a middle-aged male ESRD patient were found to be stained with black particle during his routine follow-up CAPD care [3,4]. More cases were then revealed before a national survey study reported that the incidence of this contamination was as high as 57.6 per 1,000 CAPD cases [4]. This contaminant has been hypothesized to be a potential risk for infection [3].

Without real understanding of its causal mechanism, preventive measure is not possible. Initially, iodine-based disinfectant was suspected to be the cause and attempts had therefore been made but failed to prevent the contamination [3]. As iodine and silicone are not found in normal peritoneal dialysate, we believe that the black-stained particle is a contamination during the process of peritoneal dialysis and transfer set exchange. This might be caused by clinical care provided, patient clinical condition and self-care, as well as equipment used. This might explain why attempts that focus on only one aspect of the etiology failed to prevent the contamination. The objective is to present an analysis of nurse practitioners’ perspective, which is useful for identifying potential causes of black particles.

## Findings

The causal analysis of black particle was done by Delphi technique, in which three rounds of survey were conducted among nurse practitioners. They were all certified and therefore regarded as ‘experts’ in caring for CAPD patients and should be able to reflect potential issues that might be associated with the black-stained particle incidence. The questionnaire contained 9 structural and 12 process factors, using 5-point Likert scale (1, Strongly Disagree; 2, Disagree; 3, Neither Agree or Disagree; 4, Agree; 5, Strongly Agree).

While more detailed Delphi methodology can be found elsewhere [5], the followings describe our Delphi procedure. In the first round, the questionnaire was sent to 127 PD nurses in PD centers throughout the country. Their responses were then analyzed in order to come up with an anonymous summary, which was presented in the second and third round of the questionnaire survey among 80 and 200 PD nurses who attended the 2^nd^ and 3^rd^ Annual PD Nurses Conference (PD Kem Kaeng) in October 2010 and November 2011, respectively.

Spearman’s rank correlation test was used for comparing responses of PD nurses who have seen the black particle versus those who have not whereas descriptive statistics was used for summarizing data of each round of the Delphi. This study was approved by the Institutional Review Board of the Faculty of Medicine, Chulalongkorn University (IRB No.241/54). The inform consent was signed in the invitation letter by the hospital director because the information was about the actual practice in the respective hospital but not about the nurse practitioner themselves. They were explained about their rights as a research subject and that responding to survey items would serve as their consent to participate in the research. The written informed consent was therefore not obtained from the nurses.

The response rates for the first, second, and third rounds were 73/127 (57.48%), 65/80 (81.25%), and 150/200 (75%) respectively. The nurses were 38 years of age with 16 years of nursing experience and 2.72 years of PD experience on average. Table 1 lists all potential factors for contamination from the nurse practitioner’s viewpoint, with mean scores and 95% confidence intervals in round 1, 2, and 3. The changes of means across the three rounds reflect the extent to which the respondent groups modified their responses to each of the factors whereas final interpretation would be made using the last round.

**Table 1:** Potential structural and process factors for contamination

	**Round 1**	**Round 2**	**Round 3**
Inadequate number of nephrologist	1.94 [1.58, 2.29]	2.05 [1.70, 2.40]	2.11 [1.91, 2.30]
Inadequate number of PD nurse	2.00 [1.66, 2.34]	2.65 [2.27, 3.04]	2.51 [2.29, 2.73]
Patient male gender	1.68 [1.44, 1.92]	1.79 [1.52, 2.06]	2.06 [1.89, 2.23]
Patient history of diabetes	2.61 [2.29, 2.94]	2.59 [2.26, 2.93]	2.79 [2.61, 2.97]
Patient history of peritonitis	2.68 [2.39, 2.98]	2.81 [2.49, 3.14]	3.03 [2.83, 3.22]
Care by patient’s relatives	2.16 [1.87, 2.45]	2.75 [2.42, 3.08]	2.59 [2.40, 2.77]
Use APD	1.58 [1.38, 1.78]	1.71 [1.48, 1.95]	2.60 [2.42, 2.76]
Low serum albumin	2.56 [2.24, 2.89]	2.97 [2.66, 3.28]	2.84 [2.66, 3.02]
Inadequate dialysis	2.21 [1.93, 2.48]	2.81 [2.50, 3.13]	3.00 [2.80, 3.20]
Types of Tenchkoff catheter	2.11 [1.82, 2.40]	2.32 [2.03, 2.60]	2.41 [2.23, 2.58]
Antibiotic prophylaxis	1.69 [1.44, 1.94]	1.82 [1.56, 2.09]	2.26 [2.07, 2.45]
Use of povidone-iodine for dressing	2.67 [2.33, 3.00]	3.11 [2.74, 3.47]	3.35 [3.16, 3.55]
Use of chlorhexidine for dressing	1.83 [1.60, 2.07]	2.18 [1.91, 2.45]	2.39 [2.21, 2.56]
Dressing techniques	2.32 [1.99, 2.66]	2.71 [2.39, 3.04]	2.78 [2.59, 2.97]
Lack of soaking transfer set in antiseptics	2.11 [1.80, 2.42]	2.49 [2.15, 2.84]	2.81 [2.62, 3.01]
Use of povidone-iodine during transfer set change	3.05 [2.71, 3.38]	3.50 [3.17, 3.83]	3.43 [3.24, 3.62]
Use of chlorhexidine during transfer set change	1.76 [1.53, 1.99]	2.37 [2.07, 2.67]	2.42 [2.26, 2.58]
Had dry abdomen period	2.62 [2.22, 3.03]	2.89 [2.49, 3.30]	2.76 [2.57, 2.94]
Use of Baxter (India) as dialysate	2.22 [1.92, 2.53]	2.76 [2.41, 3.11]	2.43 [2.26, 2.61]
Use of Baxter (Singapore) as dialysate	2.08 [1.81, 2.36]	2.39 [2.07, 2.70]	2.35 [2.19, 2.51]
Use of Fresenius as dialysate	1.82 [1.55, 2.09]	2.21 [1.90, 2.52]	2.26 [2.10, 2.42]
Use of GHP as dialysate	1.96 [1.58, 2.35]	2.15 [1.84, 2.46]	2.21 [2.05, 2.36]

Approximately half of the nurses in each round had seen the black particles. Using data from the third round, nurses who had and had not seen the black particles gave similar opinion regarding the causes of the contamination in general (Spearman’s rho 0.8711; p < 0.01). However, some major discrepancies (i.e. having dry abdomen period, inadequate number of PD nurse, and dressing techniques) were observed (Table 2). The findings suggested that povidone-iodine should be used with caution and that special nursing care should be provided to patients with history of peritonitis or diabetes, inadequate dialysis, and low serum albumin level.

**Table 2:** Comparison of ranks between respondents who have seen and have not seen the black particle

	**Unseen (N = 76)**	**Rank**	**Seen (N = 74)**	**Rank**
Use of povidone-iodine during transfer set change	3.37	1	3.50	1
Use of povidone-iodine for dressing	3.33	2	3.38	2
Patient history of peritonitis	3.18	3	2.86	3
Inadequate dialysis	3.16	5	2.84	4
Had dry abdomen period	2.80	10	2.72	5
Low serum albumin	2.99	7	2.69	6
Patient history of diabetes	2.93	8	2.65	7
Inadequate number of PD nurse	2.50	15	2.53	8
Lack of soaking transfer set in antiseptics	3.11	6	2.51	9
Care by patient’s relatives	2.74	11	2.43	10
Use APD	2.80	9	2.39	11
Dressing techniques	3.17	4	2.38	12
Use of Baxter (India) as dialysate	2.49	16	2.38	13
Use of Baxter (Singapore) as dialysate	2.34	18	2.35	14
Types of Tenchkoff catheter	2.53	14	2.29	15
Use of chlorhexidine during transfer set change	2.58	12	2.26	16
Use of chlorhexidine for dressing	2.54	13	2.23	17
Antibiotic prophylaxis	2.35	17	2.18	18
Use of Fresenius as dialysate	2.34	19	2.18	19
Use of GHP as dialysate	2.30	21	2.11	20
Inadequate number of nephrologist	2.12	22	2.09	21
Patient male gender	2.30	20	1.81	22

Note: Data from the third round (N = 150).

## Discussion

While black-stained particle is common in the tubes of CAPD patients, its causal mechanism has been unclear. Previous attempts to prevent this contamination that focused only on limiting the use of iodine-based disinfectant have failed to reduce the incidence [3] and more study on the causation was suggested [4].

Our study sought to understand potential practice-oriented explanation by asking nurse practitioners’ opinion. The Delphi approach helped us to improve the validity of the findings. The nurses consistently believed that the black-stained particles were caused by spilled-out povidone-iodine solution during transfer set change. Other potential causes were previous peritonitis, inadequate dialysis, low serum albumin, transfer set soaking with antiseptics, patient history of diabetes, dressing technique, and existence of dry abdomen period. This can be a good starting point for understanding the etiology and better management of this contamination.

**Figure 1:**
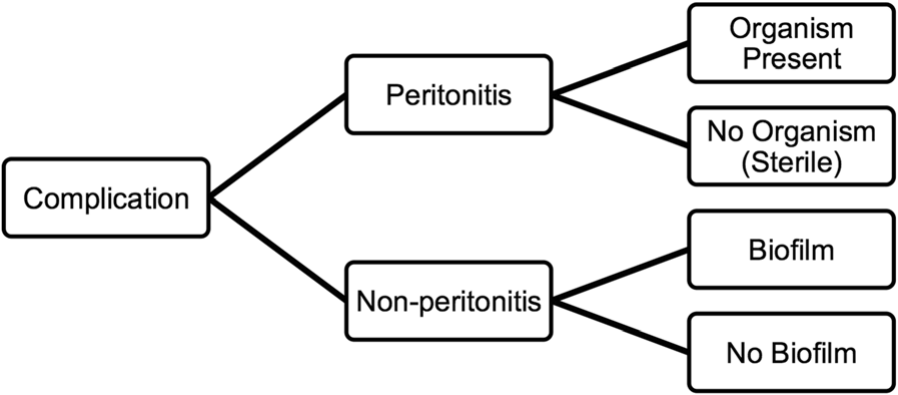
Analytical framework for etiology of the black-stained particle.

We propose that some issues must be clarified before we can conclude about the etiology of the black-stained particle (Figure 1). First, we need to assess whether the contamination is associated with peritonitis. Our study did not find adequate evidence to confirm inflammation process as required in standard diagnostic criteria [6]. In an unpublished experiment, the black particle was injected into peritoneal cavity of Wistar rat and found no significant inflammation or necrosis of visceral organs (Panomrerngsak A, et al, 2011; unpublished study). If the contamination actually causes peritonitis (but we failed to demonstrate), the second question is whether any organism is involved. If not, this contamination can be regarded as a type of sterile peritonitis [7]. Infectious peritonitis that fails to respond to antibiotic treatment should be assessed whether a biofilm is formed [8].

A number of classification systems for CAPD complications have been proposed. In 1983, Gloor et al suggested that catheter-related complications included infections, drainage failure, dialysis leak, and other [9]. In 2006, Saxena and West described five broader categories of CAPD complications; namely, infections, intra-abdominal pressure-associated, mechanical, metabolic, and miscellaneous. With relatively high incidence and potentially different management approach, we argue that the black-stained particle deserves its own category for CAPD complications.

A few limitations should be noted. The generalizability of the causal analysis using Delphi approach is limited by the knowledge and opinion of the nurse practitioners. Also, the assessment focused more on the causes that arise from practice but not other factors outside dialysis centers (i.e. medical products).

## Conclusions

Black-stained particle is a common contamination of dialysis tube in CAPD patients. This study proposed some potential determinants, most of which were relevant to care process.

## Competing interest

The results presented in this paper have not been published previously in whole or part, except in the abstract accepted for oral presentation at the 28th Annual Conference of the Royal College of Physicians of Thailand, April, 2012 and for poster presentation at the 14th Congress of the International Society for Peritoneal Dialysis, September 9-12, 2012, Kuala Lumpur, Malaysia. The authors declare that they have no competing interests.

## Authors’ contributions

WA and KP initiated the idea, collected and analyzed the data, and drafted the manuscript. TK helped to facilitate the data collection and manuscript drafting. Both authors read and approved the final manuscript.
